# *De novo* assembly of a chromosome-level reference genome of the ornamental butterfly *Sericinus montelus* based on nanopore sequencing and Hi-C analysis

**DOI:** 10.3389/fgene.2023.1107353

**Published:** 2023-03-08

**Authors:** Jingjing Li, Haiyan Wang, Jianqing Zhu, Qi Yang, Yang Luan, Leming Shi, José Arturo Molina-Mora, Yuanting Zheng

**Affiliations:** ^1^ State Key Laboratory of Genetic Engineering, School of Life Sciences and Human Phenome Institute, Fudan University, Shanghai, China; ^2^ Grandomics Biosciences Institute, Wuhan, China; ^3^ Shanghai Zoological Park, Shanghai, China; ^4^ Shanghai Jiao Tong University School of Medicine, Shanghai, China; ^5^ Cancer Institute, Shanghai Cancer Center, Fudan University, Shanghai, China; ^6^ Centro de Investigación en Enfermedades Tropicales, Facultad de Microbiología, Universidad de Costa Rica, San José, Costa Rica

**Keywords:** sericinus montelus, aristolochic acid tolerance, genome assembly, oxford nanopore sequencing, hi-c

## Abstract

*Sericinus montelus* (Lepidoptera, Papilionidae, Parnassiinae) is a high-value ornamental swallowtail butterfly species widely distributed in Northern and Central China, Japan, Korea, and Russia. The larval stage of this species feeds exclusively on *Aristolochia* plants. The *Aristolochia* species is well known for its high levels of aristolochic acids (AAs), which have been found to be carcinogenic for numerous animals. The swallowtail butterfly is among the few that can feed on these toxic host plants. However, the genetic adaptation of *S. montelus* to confer new abilities for AA tolerance has not yet been well explored, largely due to the limited genomic resources of this species. This study aimed to present a chromosome-level reference genome for *S. montelus* using the Oxford Nanopore long-read sequencing, Illumina short-read sequencing, and Hi-C technology. The final assembly was composed of 581.44 Mb with an expected genome size of 619.27 Mb. Further, 99.98% of the bases could be anchored onto 30 chromosomes. The N50 of contigs and scaffolds was 5.74 and 19.12 Mb, respectively. Approximately 48.86% of the assembled genome was suggested to be repeat elements, and 13,720 protein-coding genes were predicted in the current assembly. The phylogenetic analysis indicated that *S. montelus* diverged from the common ancestor of swallowtails about 58.57–80.46 million years ago. Compared with related species, *S. montelus* showed a significant expansion of P450 gene family members, and positive selections on *eloa*, *heatr1*, and *aph1a* resulted in the AA tolerance for *S. montelus* larva. The *de novo* assembly of a high-quality reference genome for *S. montelus* provided a fundamental genomic tool for future research on evolution, genome genetics, and toxicology of the swallowtail butterflies.

## Introduction

**FIGURE 1:**
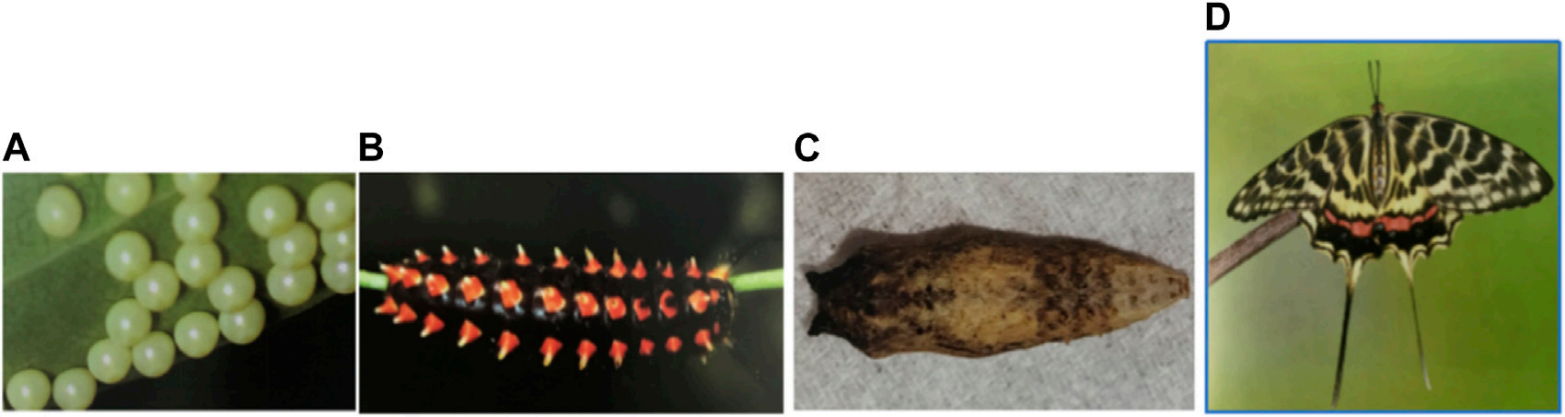
Four phases of the complete life history of *S. montelus*: egg, larva, pupa, and adult.

Butterflies account for the high biodiversity of terrestrial organisms, with more than 18,000 species recorded worldwide (Espeland et al., 2018). Butterflies develop a life cycle through a complete metamorphosis spanning four stages: egg, larva, pupa, and adult (Figure 1). More than half a century ago, butterflies were widely used as important model species to investigate the impact of habitat destruction, pest control, and climatic changes. Further, the study of butterflies has progressed tremendously in other diverse fields: evolution, embryology, mimicry, toxicology, genetics, population dynamics, and biodiversity conservation (Heikkila et al., 2012; Kawahara and Breinholt, 2014).

*Sericinus montelus* (Lepidoptera, Papilionidae, Parnassiinae) is a common species of swallowtail butterfly with high ornamental values native only to Northern and Central China, Japan, Korea, and Russia (Li et al., 2016). In the last few years, *S. montelus* was used to study its four stages of life and aspects such as natural history, breeding, mobility, climate change, and habitat loss (Li et al., 2016). The larvae of *S. montelus* are monophagous insects that feed on *Aristolochia contorta* (Li et al., 2016). However, the host plant notoriously contains toxic substances such as aristolochic acid (AA), which is carcinogenic to many animals (Arlt et al., 2002; Chen et al., 2012). In mammal models, AAs are metabolically converted into form reactive intermediates, and these intermediates have the potential to bind to DNA and exert mutagenic and carcinogenic effects (Lebeau et al., 2001).

Many biological aspects of *S. montelus* remain unclear to date. For example, why do the *S. montelus* larvae only feed on a single host plant *Aristolochia*; which functional genes and metabolic pathways are essential for *S. montelus* to be AA tolerant; and how *S. montelus* is tolerant to the genotoxicity of AAs. Indeed, comprehensive genomic data analyses are needed to address these questions. Therefore, the assembly of a high-quality chromosome-level reference genome for *S. montelus* can provide a genomic tool to fill this gap.

Whole-genome sequencing provides a high-resolution and comprehensive collection of an individual’s genetic variations for target species at the base-by-base level (Ng and Kirkness, 2010). The sequencing and assembly of a complex genome for insect species rapidly and in a cost-effective manner has become possible with the development of third-generation sequencing technology (Oxford Nanopore and PacBio sequencing technologies) (Lu et al., 2019; Yang et al., 2020). Moreover, high-throughput chromosome conformation capture (Hi-C) is an effective technology (Belaghzal et al., 2017) to identify genome-wide interactions between and within chromosomes. The Hi-C technology is a powerful tool to improve the genome assembly to the chromosome level (Belaghzal et al., 2017).

This study reported a high-quality chromosome-level reference genome of *S. montelus* using the Oxford Nanopore long reads, Illumina short reads, and Hi-C scaffolding technology. This was the most contiguous reference genome generated for the swallowtail butterflies so far. Comparative genome analyses provided evidence to identify candidate functional genes and metabolic pathways related to AA tolerance. The genome of S. montelus reported in this study might provide important data for further investigations on the evolution biology of the swallowtail butterflies, complexity of host-plant shifts, and genomic aspects of key affiliated genes for the adaptation to AA tolerance.

## Materials and methods

### DNA collection and sequencing

A fifth instar larval stage of *S. montelus* was collected from Beijing mountainous area. High–molecular weight genomic DNA was extracted using the SDS method, followed by purification with Qiagen genomic kit following the standard protocol. DNA purity was then assessed based on OD 260/280 and OD 260/230 ratios detected using NanoDrop One/OneC Microvolume UV-Vis Spectrophotometer (Thermo Fisher Scientific, USA). DNA concentration was measured using a Qubit 3.0 Fluorometer (Invitrogen, USA).

Illumina library with an insert size of 350 bp was prepared using a TruSeq Nano DNA HT sample preparation kit (Illumina, USA). DNA fragments were then blunted with an A-base overhang and ligated to sequencing adapters for Illumina sequencing with further polymerase chain reaction (PCR) amplification. Finally, PCR products were purified (AMPure XP system), and libraries were analyzed for size distribution using an Agilent 2100 Bioanalyzer and quantified using real-time PCR. After the aforementioned quality control steps, the library was sequenced on an Illumina NovaSeq 6000 platform in a paired-end sequencing model. Further, 2 µg of DNA was used for Nanopore library construction. The size selection (20 kb) was performed using a BluePippin system (Sage Science, USA). Next, both ends of DNA fragments were repaired and an A-ligation reaction was conducted using an NEBNext Ultra II End Repair/dA-tailing Kit (Cat# E7546). The adapter in the LSK109 kit was used for further ligation reaction, and a Qubit 3.0 Fluorometer (Invitrogen, USA) was used to quantify the size of library fragments. Sequencing was then performed on a Nanopore PromethION (Oxford Nanopore Technologies, United Kingdom) at Grandomics (Wuhan, China).

### Hi-C library construction

A Hi-C library was constructed from a fifth instar larval stage following the previously reported protocols (Lafontaine et al., 2021). In brief, freshly harvested tissues were cut into 2-cm pieces and vacuum infiltrated in nuclei isolation buffer supplemented with 2% formaldehyde. Glycine and additional vacuum infiltration were used to stop the process. The fixed tissue was then grounded to a powder before resuspending it in a nuclei isolation buffer to obtain a nuclear suspension. One hundred units of DpnII were used to digest the purified nuclei, followed by marking with biotin-14-dCTP. Biotin-14-dCTP from non-ligated DNA ends was removed owing to the exonuclease activity of T4 DNA polymerase. The ligated DNA was sheared into 300–600 bp fragments and then blunt-end repaired and A-tailed, followed by purification through biotin-streptavidin-mediated pull-down. Finally, the Hi-C library was quantified using the aforementioned steps and then sequenced at Illumina NovaSeq 6000 platform.

### Genome size estimation

Raw data generated from Illumina sequencing were quality controlled using fastp v0.20.0 (Chen et al., 2018), and low-quality reads, adapters, and reads containing N were filtered. Specifically, the reads were filtered under the following conditions: 1) reads with ≥10% unidentified nucleotides N); 2) reads with >10 nucleotides aligned to the adapter, allowing ≤10% mismatch; 3) reads with >50% bases having Phred quality <5; 4) putative PCR duplicates generated by PCR amplification removed in the library construction process (reads 1 and 2 of two paired-end reads that were completely identical). A total of 100,000 reads were selected randomly and compared against the sequences deposited in the NT database (nucleotide sequence database) to verify the presence of contamination (Sayers et al., 2022) using blastn v2.12.0+ (Mount, 2007). After quality control, the clean reads were used to count the number of 17-mers using jellyfish2 v2.3.0 (Marcais and Kingsford, 2011) and the distributions were then used to estimate the genome size with GenomeScope v2.0b (Vurture et al., 2017).

### Chromosome-level genome assembly and validation

The raw reads of Oxford Nanopore were corrected using NextDenovo v2.5.0 (https://github.com/Nextomics/NextDenovo) with seed length cutoff set to 23 kb, and the corrected reads were then assembled using SMARTdenovo v1.0.0 (Liu et al., 2021) with parameters “-k 21 -J 3000” to generate the primary contigs. Three rounds of iterative polishing using Nextpolish v1.2.3 (Hu et al., 2020) were implemented based on Oxford Nanopore reads and Illumina short reads to enhance the continuity and base accuracy of the assembly. Subsequently, the polished assembly was decontaminated. The reads aligned in terms of depth and GC content were calculated on contigs using a 10-kb window bin. The contigs having 80% bins with GC less than 0.2 or greater than 0.6 were selected. Then, the contigs were aligned to the NT database (ftp://ftp.ncbi.nlm.nih.gov/blast/db/), and contigs aligned to bacterial and viral species were removed. After genome decontamination, the assembly was used to evaluate the quality of the Hi-C library and valid read pairs were identified following the Hic-Pro pipeline v2.8.1 (Servant et al., 2015). These valid read pairs were then used to calculate the interactions between every two regions of the whole genome to generate an interaction matrix, which was used to cluster, order, and orient the contigs onto a designated number of chromosomes using LACHESIS version-201701 (Burton et al., 2013) with default settings. In the LACHESIS step, manual curation was used to reduce assembly errors based on the whole-genome interaction matrix signal. In the contig clustering process, contigs were split into a 50-kb interaction matrix. The contigs were interrupted by manual curation when the interaction bins of one contig were clustered into different chromosomes. In contig ordering and orientation steps, manual curation was used to ensure that the contig was in the right position in chromosomes based on the interaction matrix score.

The quality of the assembly was validated using different strategies based on contiguity, correctness, and completeness (3C) criteria (Molina-Mora et al., 2020). For contiguity, the statistics about total assembled length, total number of contigs and scaffolds, and contig and scaffold N50 (N50 value was calculated at the median of the total length by sorting all contigs with length) were calculated. Three analyses were performed to assess completeness: i) BUSCO v5.1.3 (Manni et al., 2021) with insect_odb10 datasets was used to evaluate the assembly of expected genes; ii) RNA sequencing (RNA-seq) reads were mapped back to the genome with hisat2 v2.1.0 (Kim et al., 2019) with default settings to evaluate the mapping rate and coverage of the gene region of the assembly; iii) valid Hi-C reads were mapped back to the pseudo-chromosomes, and the interaction matrix between 100-kb bins was calculated based on the mapping results; iv) Nanopore long reads and Illumina short reads were, respectively, mapped back to the assembly using minimap2 v2.17-r954-dirty (Li, 2018) and bwa v0.7.16a-r1181 (Li and Durbin, 2009) to calculate the mapping rate and coverage; and iv) DepthSizer v1.6.3 (Chen et al., 2022) was used to establish the collapsed repeats in the assembly and the completeness of the genome assembly. In the last case, the mapping results were used to evaluate the genome’s accuracy or correctness. Finally, the mapped reads were used to detect variants by employing samtools v1.13 (Li et al., 2009) and bcftools v1.9 (Li, 2011), and homozygous SNPs and indels were considered as genomic errors.

### RNA collection and sequencing

RNA-seq was achieved for four samples representing four stages of *S. montelus* (egg, larva, pupa, and adult; Figure 1) to assist in the gene predictions in the genome annotation step. Total RNA from each sample was collected using an RNeasy Plus Mini Kit (Qiagen, Germany). The RNA purity was checked using a NanoDrop One/OneC spectrophotometer (Thermo Fisher Scientific). RNA degradation and contamination were monitored using 1% agarose gels. The RNA concentration was measured using a Qubit RNA Assay Kit of a Qubit 3.0 Fluorometer (Life Technologies, CA, USA). The RNA integrity was assessed using an RNA Nano 6000 Assay Kit of a Bioanalyzer 2100 system (Agilent Technologies, CA, USA). The RNA quality for the RNA samples with an integrity value more than 8.0 was used for constructing the cDNA library. The paired-end library was prepared using an Illumina TruSeq Sample Preparation Kit and sequenced on a NovaSeq6000 platform (Illumina). The raw data were filtered by removing reads containing adapters, Ns, and low-quality reads using fastp v0.20.0 (Chen et al., 2018).

### Genome annotation

The repeat sequences, including tandem repeat and transposable elements (TEs) residing in the genome, were investigated. The tandem repeats were identified using GMATA v2.2 (Wang and Wang, 2016) and Tandem Repeats Finder (TRF) v4.07b (Benson, 1999). Briefly, GMATA (Wang and Wang, 2016) was used to identify simple sequence repeats (SSRs), and TRF (Benson, 1999) was used to recognize all tandem repeat elements in the whole genome. The TEs residing in the genome were identified using a combination of *ab initio* and homology-based methods. To this end, MITE-hunter (Han and Wessler, 2010) and RepeatModel2 v1.0.11 (Flynn et al., 2020) were used to identify possible TEs with default parameters and create a repeat library. The library was then classified according to homologous deposits in the Repbase v14.02 (Bao et al., 2015). RepeatMasker v1.331 (Tarailo-Graovac and Chen, 2009) was applied to search for known and novel TEs by mapping sequences against the *de novo* repeat and Repbase library for further identification of the repeats throughout the genome. Overlapping TEs belonging to the same repeat class were collated and combined.

The protein-coding genes were predicted by integrating *ab initio*, homology-based, and RNA-seq-based strategies. For homology prediction, GeMoMa v1.6.1 (Keilwagen et al., 2019) was used to determine the gene structure based on the alignments of homologous peptides from five related species, including *Bombyx mori* (GCF_014905235.1), *Danaus plexippus* (GCF_009731565.1), *Papilio glaucus* (GCA_000931545.1), *Papilio machaon* (GCF_912999745.1), and *Papilio xuthus* (GCF_000836235.1) mapped back to the assembly. For RNA-seq-based gene prediction, clean RNA-seq data were mapped back to the reference genome using STAR v2.7.3a (Dobin et al., 2013). The transcripts were then assembled using Stringtie v1.3.4d (Pertea et al., 2015), and open reading frames (ORFs) were predicted using PASA v2.3.3 (Haas et al., 2008). The PASA results were also used as a training set to train profiles for *de novo* prediction tools including Augustus v3.3.1 (Stanke et al., 2006) and SNAP (Korf, 2004). Subsequently, Augustus and SNAP were used to predict possible protein-coding regions with default parameters. Finally, EVidenceModeler v1.1.1 (EVM) (Haas et al., 2008) was used to produce an integrated gene set, and genes harboring TEs were removed using the TransposonPSI package (http://transposonpsi.sourceforge.net/). The miscoded genes were further filtered. The untranslated regions (UTRs) and alternative splicing regions were determined using PASA based on RNA-seq assemblies. The longest transcripts were retained for each locus, and the regions outside of the ORFs were designated UTRs.

The non-coding RNAs (ncRNA), including micro RNAs (miRNAs), ribosomal RNAs (rRNAs), small nuclear RNAs (snRNAs), and transfer RNAs (tRNAs), were determined with the Rfam database (release 13.0) (Kalvari et al., 2017) using Infernal v1.1.3 software (Nawrocki, 2014). tRNA was identified using tRNAscan-SE v2.0 software (Chan et al., 2021). The subunits of rRNA were predicted using RNAmmer v1.2 software (Lagesen et al., 2007).

The functional annotation of the predicted protein-coding genes was carried out by aligning them with homologs deposited in public databases. Briefly, the predicted proteins were searched against homologs deposited in SwissProt (https://ftp.uniprot.org/pub/databases/uniprot/), NR (ftp://ftp.ncbi.nlm.nih.gov/blast/db/FASTA/nr.gz), KEGG (https://www.kegg.jp/kegg/download/), and KOG (http://ftp//ftp.ncbi.nih.gov/pub/COG/KOG/) using the blastall v2.2.26 blastp model (Mount, 2007) with an E-value cutoff of 1e-05. Descriptions, gene ontologies, and KEGG pathways were extracted from the top hits.

### Gene family and phylogenetic analysis

The orthogroups between *S. montelus* and relative species that seemed to originate from common ancestor sequences were investigated. To this end, genome sequences, GFF3 file, CDS, and protein sequences of relative species, including *P. xuthus*, *Papilio polytes*, *P. machaon*, *P. glaucus*, *Papilio binaor*, *Melitaea cinxia*, *Junonia coenia*, *Heliconius melpomene*, *D. plexippus*, and *B. mori*, were downloaded from NCBI (Kitts et al., 2015). For each species, the longest transcript for each gene was extracted from the genome and GFF3 file, and miscoded genes and genes exhibiting premature termination were discarded by checking whether the coding sequence accorded with the gene model. These genes whose CDS did not begin with a start codon or did not end with a stop codon, or whose CDS length was not a multiple of three, or genes with a stop codon in the middle of the CDS were discarded. Following the extraction of protein sequences, they were aligned in pairs using the blastall blastp model (Mount, 2007), with an E-value cutoff of 1e-05. The inter-genome orthologous, intra-genome paralogous, and single-copy gene pairs were further identified using OrthMCL v2.0.9 (Li et al., 2003).

After identifying orthologous gene sets, a molecular phylogenetic analysis was performed using the shared single-copy genes. Briefly, the coding sequences were extracted from the single-copy families, and each orthogroup was globally aligned using Mafft v7.313 (Katoh et al., 2002) with default settings. Poorly aligned sequences were then eliminated using Gblocks v0.91b (Castresana, 2000), and the GTR + GAMMA substitution model of RAxML v8.2.10 (Stamatakis, 2015) was used for the phylogenetic tree construction with 1000 times of ultrafast bootstrap resampling. The generated tree file was displayed using Figtree v1.4.4 (http://tree.bio.ed.ac.uk/software/figtree/). MCMCTREE v4.9e included in the PAML4 (Yang, 2007) package was used to estimate the divergent time based on the phylogenetic tree. The calibration times were obtained from the TimeTree database (http://www.timetree.org/) by placing soft bounds at the split node of *B. mori* (114 Mya), *P. glaucus*–*J.coenia* (76–106 Mya), and *D. plexippus*–*J. coenia* (77–107 Mya) (Kumar et al., 2017).

Significant expansion or contraction of specific gene families is often associated with the adaptive divergence of closely related species. Hence, CAFÉ v4.0 (De Bie et al., 2006) was used to perform a gene family expansion and contraction based on the results of OrthMCL and estimated divergence times. The enrichment analysis for the expanded gene family members was implemented using clusterProfiler v4.0 (Wu et al., 2021). After the identification, the gene lists were analyzed with functional enrichment using GO terms and KEGG pathways. The significantly enriched determinants were extracted individually for comparison against model organisms.

### Positive selection analysis

According to the neutral theory of molecular evolution, the ratio of non-synonymous substitution rate (*K*_a_) and synonymous substitution rate (*K*_s_) of protein-coding genes can be used to identify genes showing the signatures of natural selection. Therefore, the branch-site model in the codeml model (Yang, 2007) was used to estimate possible adaptations that resided in the proteins in the *S. montelus* genome with orthologous genes. Likelihood ratio tests were performed between the null hypothesis (model = 2, NSsites = 2, fix_omega = 1, omega = 1) and alternative hypothesis (model = 2, NSsites = 2, fix_omega = 0, omega = 1.5) to determine the fitness differences between these two models. Genes with a *p*-value < 0.05 under the branch-site model were considered positively selected genes. The *p* values were also adjusted using the Benjamini–Hochberg method (Benjamini and Hochberg, 1995), given the multiple tests. The process was repeated twice for each gene to test the convergence of the HMM models. The three-dimensional structure for the two candidates of positively selected genes was predicted using AlphaFold2 (Bouatta et al., 2021) and visualized using PyMOL (www.pymol.org/pymol).

The function enrichment analysis of positive selection genes in GO terms and KEGG pathways was conducted using clusterProfiler v4.0. The significant enrichment pathways were defined with a *p*-value < 0.05. For RNA-seq data, the value of fragments per kilobase per million mapped reads (FPKM) was used to characterize the gene expression profile at a different stage with the stringtie v1.3.4 software, and heatmap was plotted with HeatMapper (http://www.heatmapper.ca/expression/).

## Results and discussion

### Chromosome-level genome assembly

A total of 25.9-Gb Illumina short reads (Table 1) were produced and used to estimate the size and heterozygosity of the *S. montelus* genome. The main peak was 33 according to the distribution of 17-mers (Supplementary Figure S1), and the deduced size and heterozygosity of the *S. montelus* genome were approximately 619.27 Mb and 0.8% (Supplementary Table S1), respectively.

**TABLE 1:** Raw data of *S. montelus* generated in the present study using Nanopore and Illumina technology.

Platform	Aim	Read length	Total length (Gb)
Nanopore PromethION (WGS)	*De novo* genome assembly	22 kb (mean)	55.73
Illumina NovaSeq (WGS)	Genome survey and polish	2 × 150 bp	25.91
Illumina NovaSeq (WGS)	Hi-C scaffolding	2 × 150 bp	90.95
Illumina NovaSeq (RNA-Seq)	Gene annotation (egg)	2 × 150 bp	9.68
Illumina NovaSeq (RNA-Seq)	Gene annotation (larva)	2 × 150 bp	12.13
Illumina NovaSeq (RNA-Seq)	Gene annotation (pupa)	2 × 150 bp	8.97
Illumina NovaSeq (RNA-Seq)	Gene annotation (adult)	2 × 150 bp	11.10

Subsequently, more than two million Oxford Nanopore long reads, approximately 55.7 Gb and representing nearly 100× of the estimated genome size, were generated and used for *de novo* genome assembly. Briefly, the long reads were first self-corrected using Nextdenovo and then assembled into primary contigs using SMARTdenovo. Considering the high error rate of Oxford Nanopore long reads, the consensus of the primary contigs was then iteratively improved using the long reads and Illumina short reads for three rounds. After genome decontamination, the final genome size was nearly 581.4 Mb, with a contig N50 of approximately 5.74 Mb (Table 2).

**TABLE 2:** Assessment of the *de novo* assembly and protein-coding gene annotation of *S. montelus*.

	Parameter	Value
Genome assembly	Chromosome numbers (2n)	60
	Estimated genome size (Mb)	619
	Assembled genome size (Mb)	581
	Longest scaffold (bp)	23,928,049
	Number of scaffolds	74
	N50 of scaffolds (bp)	19,117,648
	Longest contig (bp)	14,777,801
	Number of contigs	319
	N50 of contigs (bp)	5,744,342
	GC rate (%)	35.9
	Genome BUSCO (%)	99.4
Genome annotation	Number of protein-coding genes	13,720
	Mean gene length (bp)	16,360
	Mean CDS length (bp)	1,502
	Mean exon/intron length (bp)	207/2,373
	Mean exon per gene	7
	Total size of TEs (bp)	284,101,946
	TEs in genome (%)	48.9
	Gene BUSCO (%)	94.7
	Number of rRNAs	148
	Number of miRNAs	194
	Number of tRNAs	3,326
	Number of snRNAs	166

Karyotype is a written proof of chromosome numbers of what a cytogeneticist observes from species. Hi-C is a remarkable technology for genome scaffolding. It is a chromosome conformation capture method used to detect genome-wide chromatin interactions in many plant and animal genomes. More than 600 million of Illumina short reads (approximately 90.9 Gb representing about 150× of the estimated genome size) were generated (Hi-C strategy) to scaffold the contigs. After quality control and filtering, 152,896,732 uniquely and validly mapped paired-end reads were retained and used to construct the Hi-C interaction matrix. The chromosome number between 28 and 32 was used with Lachesis and SALSA to guide the genome scaffolding. Finally, the Hi-C matrix map showed that 30 can clustered succeed and each cluster had intra- and interchromosomal matrix data. Based on the matrix, 275 contigs of 566 Mb, representing more than 99.9% of the assembly, were orientated and anchored to 30 pseudo-chromosomes (Supplementary Table S2). The pseudo-chromosome LG04 was identified as a Z chromosome with collinear blocks.

This strategy was followed because of the high error rate (∼15%) of Nanopore reads, in which the primary assembly contig assembled using SMARTdenovo was polished by combining with high-accuracy Illumina reads (Ge et al., 2019). Further, Hi-C is an impressive technology for genome scaffolding. It is a chromosome conformation capture method used to detect genome-wide chromatin interactions in many plant and animal genomes. It is widely applied in improving the *de novo* assembled contigs into chromosome-level genome assembly, as previously reported (Yang et al., 2020).

**FIGURE 2:**
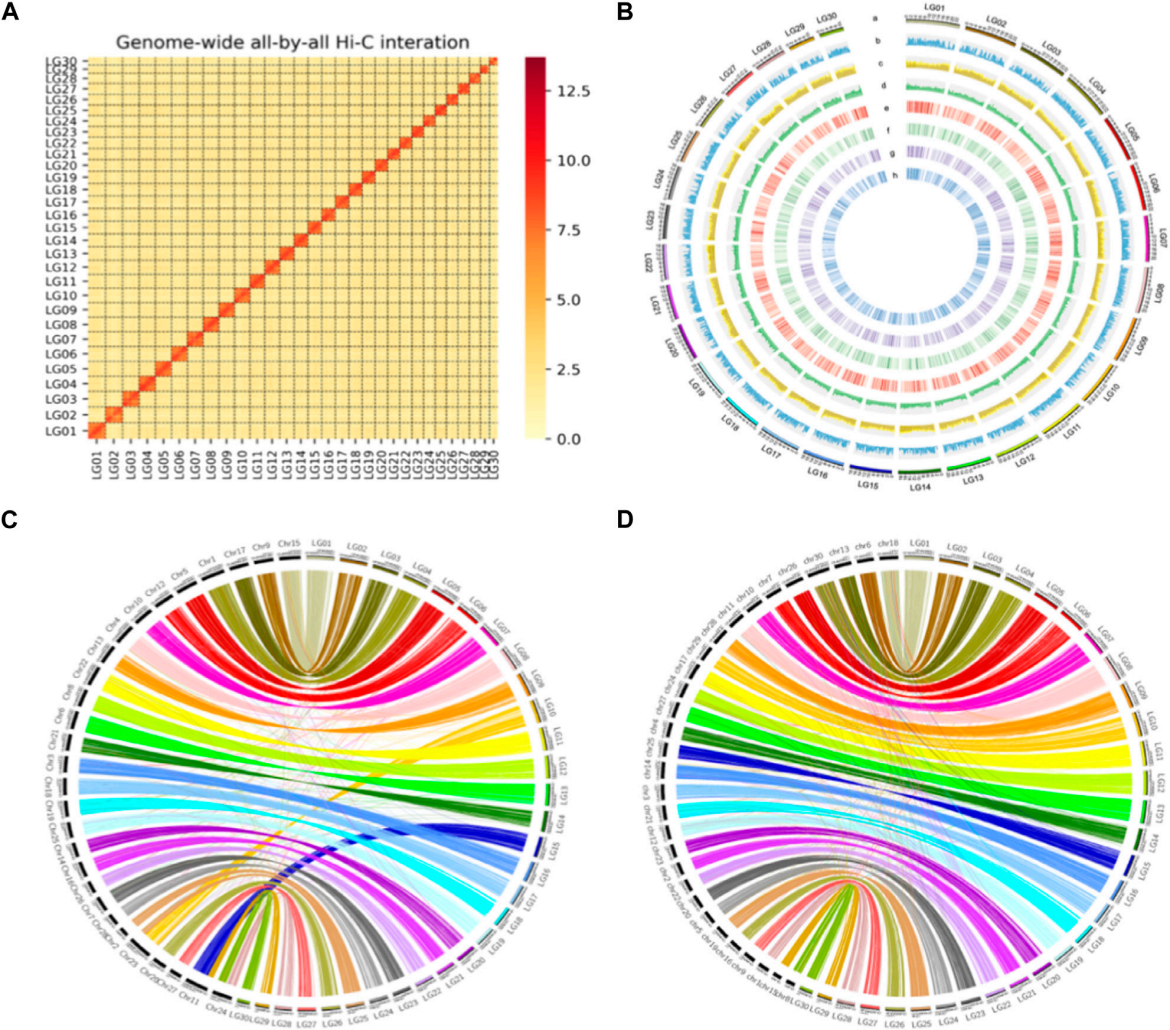
High quality *de novo* assembly of *S. montelus*. **(A)** Interaction heatmap between any two regions of the whole genome intra- and interchromosome with Hi-C data. **(B)** Whole-genome feature distribution of *S. montelus* with 1-Mb-wide bins. **a**, Chromosome (30 chromosomes); **b**, gene density (13,720 protein-coding genes); **c**, repeat density (repeat content 48.9%); **d**, GC content distribution (average GC content 35.9%); **e**, egg stage gene expression (75% of genes have expression); **f**, larva stage gene expression (84% expression); **g**, pupa stage gene expression (77% expression); **h**, adult stage gene expression (86% expression). **(C)** Protein-coding gene synteny between *S. montelus* (right) and *Bombyx mori* (left). **(D)** Protein-coding gene synteny between *S. montelus* (right) and *P. binaor* (left).

The quality of the final assembly was estimated using different strategies. For contiguity, the contig N50 and scaffold N50 were 5.74 and 19.12 Mb (Table 2), respectively, with 99.4% complete BUSCOs in the final genome. For completeness, the size of the final chromosome-level genome was about 581.44 Mb, which represented nearly 93.9% of the estimated genome size using GenomeScope and 98.94% of the estimated genome size (587.66 Mb) using DepthSizer with a combined sequencing length and the single-copy read depth software. More than 98.84% of the whole-genome sequencing short reads were mapped back to the assembly (Supplementary Table S3), and the percentage of homozygous alternative bases was estimated to be less than 7.6e-6 (Supplementary Table S4) as part of the correctness assessment. Generally, these bases indicate errors since normal alternatives should be heterozygous. Also, for completeness, short reads of RNA-seq for the four different phases, including egg, larva, pupa, and adult, were mapped back to the assembly, and the mapping rate ranged from 95.24% to 96.89% (Supplementary Table S3). The completeness using BUSCO showed that nearly 99.4% of the complete benchmark genes could be identified in the assembly, within which about 98.0% and 1.4% were single-copy and duplicated, respectively (Supplementary Figure S2). Only 0.5% of the benchmark genes were missed in the assembly. All short reads from the Hi-C library were mapped back to the final assembly, and the results showed that the interactions between nearby regions were more intensive than those between distant ones (Supplementary Figure S3), conforming to the premise of Hi-C scaffolding. Furthermore, a consistent synteny was found between the assemblies of *S. montelus* and the relative species *B. mori* and *P. binaor* (Figure 2).

Compared with the *de novo* genome assemblies generated for butterflies (Table 3), the quality of the present assembly was one of the best (Lu et al., 2019; Yang et al., 2020). Briefly, a total of 55.7 and 30.5 Gb raw data were generated for the Chinese peacock butterfly *Papilio bianor* and dead-leaf butterfly *Kallima inachus* and used for *de novo* assembly, and the final contig N50 was 5.5 and 3.3 Mb, respectively (Lu et al., 2019; Yang et al., 2020). The BUSCO assessments showed that approximately 96.3% and 97.3% of complete benchmark genes were recovered for the two species, respectively (Luan et al., 2015; Turi et al., 2018). For *S. montelus*, 55.7 Gb raw data were used for the *de novo* assembly, and the contig N50 and BUSCO assessments reached 5.7 Mb and 99.4%, respectively. The high-quality genome assembly of *S. montelus* might provide a basic understanding of genome evolution, toxicology, and AA tolerance in future analysis.

**TABLE 3:** Comparison of basic characteristics of *S. montelus* genome assembly and annotation with published butterfly genomes.

Species	Genome size (Mb)	Heterozygosity (%)	N50^a^ (Mb)	BUSCO (%)	Repeats (%)	Gene number (k)	CDS length (mean)	Sequencing technology
*S. montelus*	581	0.8	5.7	99.4	48.9	13.7	1,502	Nanopore
*P. bianor*	421	1.8	5.5	96.3	55.3	15.4	1,383	PacBio
*K. inachus*	569	NA	3.3	97.3	49.9	15.3	1,399	PacBio
*P. polytes*	227	NA	3.7	91.8	23.8	12.2	1,631	NGS
*P. xuthus*	244	1	6.2	97.6	22.4	13.1	1,580	NGS
*P. machaon*	281	1.2	1.2	95.5	22.3	15.5	1,350	NGS
*P. memnon*	233	NA	5.5	96.6	22.5	12.4	1,560	NGS
*P. glaucus*	375	2.3	0.2	95.5	22.0	15.7	1,216	NGS
*D. plexippus*	249	0.6	0.7	98.0	17.2	15.1	1,383	NGS
*H. melpamene*	274	NA	0.2	95.6	32.8	12.8	1,362	NGS
*M. cinxia*	390	NA	0.1	83.0	27.5	16.7	959	NGS

^a^N50, contig N50 for Nanopore and PacBio platform, and scaffold N50 for NGS, platform.

NA, not available in the reference.

### Identification of repeats and protein-coding genes

A total of 284.1 Mb accounting for approximately 48.9% of the whole assembly were identified as repeat elements by adopting both the *de novo* and homology-based strategies (Table 3). Generally, the type I TE long interspersed nuclear element (LINE) was the most represented in the genome, and about one-fifth of the genome was composed of LINEs (Supplementary Table S5). The second most represented TEs residing in the genome was a long terminal repeat, accounting for nearly one-tenth of the genome. The other most represented REs were DNA repeats and short interspersed nuclear elements, which accounted for about 8.8% and 3.7% of the genome, respectively.

A total of 13,720 protein-coding genes were identified in the genome by combining the results of three strategies, including the *de novo*, homology-based, and expression-based gene prediction methods. Of these, 12,422 genes (representing 90.5% of total genes) were annotated by at least one database (Table 4). Specifically, the descriptions were obtained for 12,422 and 9,955 genes from Nr and SwissProt, which accounted for nearly 90.1% and 72.6% of total genes, respectively. A total of 8,518 genes were annotated by KOG functional protein database, 6,382 genes by GO, and 6,861 genes by KEGG (Table 4). The ncRNA was predicted using the published database of Rfam (Daub et al., 2015). A total of 148 rRNAs, 194 miRNAs, 3,326 tRNAs, and 166 snRNAs were identified (Supplementary Table S6).

**TABLE 4:** Annotation summary of the predicted protein-coding genes residing in the *S. montelus* genome in the SwissProt, KOG, KEGG, GO, and NR databases.

Database	Number of genes annotated	Percentage (%)
SwissProt	9,955	72.6
KOG	8,518	62.1
KEGG	6,861	50.0
GO	6,382	46.5
NR	12,360	90.1
Total	12,422	90.5

Regarding the content of repeat sequences, the number of elements residing in *S. montelus* genome was similar to that of *K. inachus* (48.9% vs. 49.9%), slightly less than that of *P. bianor* (55.3%) but much higher than that of *P. xuthus* (22.4%), *P. machaon* (32.3%), *P. polytes* (34.0%), and others (Schmeiser et al., 2009; Stiborova et al., 2011) (Table 3). In *S. montelus*, 13.7 k genes were identified. Compared with other species, the gene number for our model was less than that of *K. inachus* (15.3 k), *P. bianor* (15.4 k), and *P. machaon* (15.5 k), but more than that of *P. xuthus* (13.1 k) and *P. polytes* (12.2 k). The *de novo* genome assemblies of *S. montelus*, *K. inachus*, and *P. bianor* were all based on the long-read sequencing technologies, but short reads were used for the other species such as *P. xuthus*, *P. machaon*, and *P. polytes*. Thus, it seemed that long-read sequencing technologies recovered more repeats residing in the genomes that short-read technologies might have expectedly missed. The long-read sequencing technologies had the advantage of resolving repetitive regions in the *de novo* assembly. For example, in a recent study, the Telomere-to-Telomere (T2T) Consortium used long-read sequencing technologies to finish a gapless human T2T-CHM13 genome and addressed 8% of the genome with the most repeated regions (Nurk et al., 2022).

On the contrary, frameshifts may occur for long-read raw assemblies because of the high error rate compared with Illumina assemblies (Koren et al., 2019). This situation can affect gene prediction and total gene counts (Molina-Mora et al., 2020). To address this, polishing was used as a key step to improve the genome base-pair accuracy to QV50 by mapping long and short reads back to the assembly. Thus, after the short-read correction process, total gene numbers and average CDS length resulted in similar counts and lengths for all the compared genomes, independently of the sequencing technology.

### Possible adaptive gene family expansions of *S. Montelus*

**FIGURE 3:**
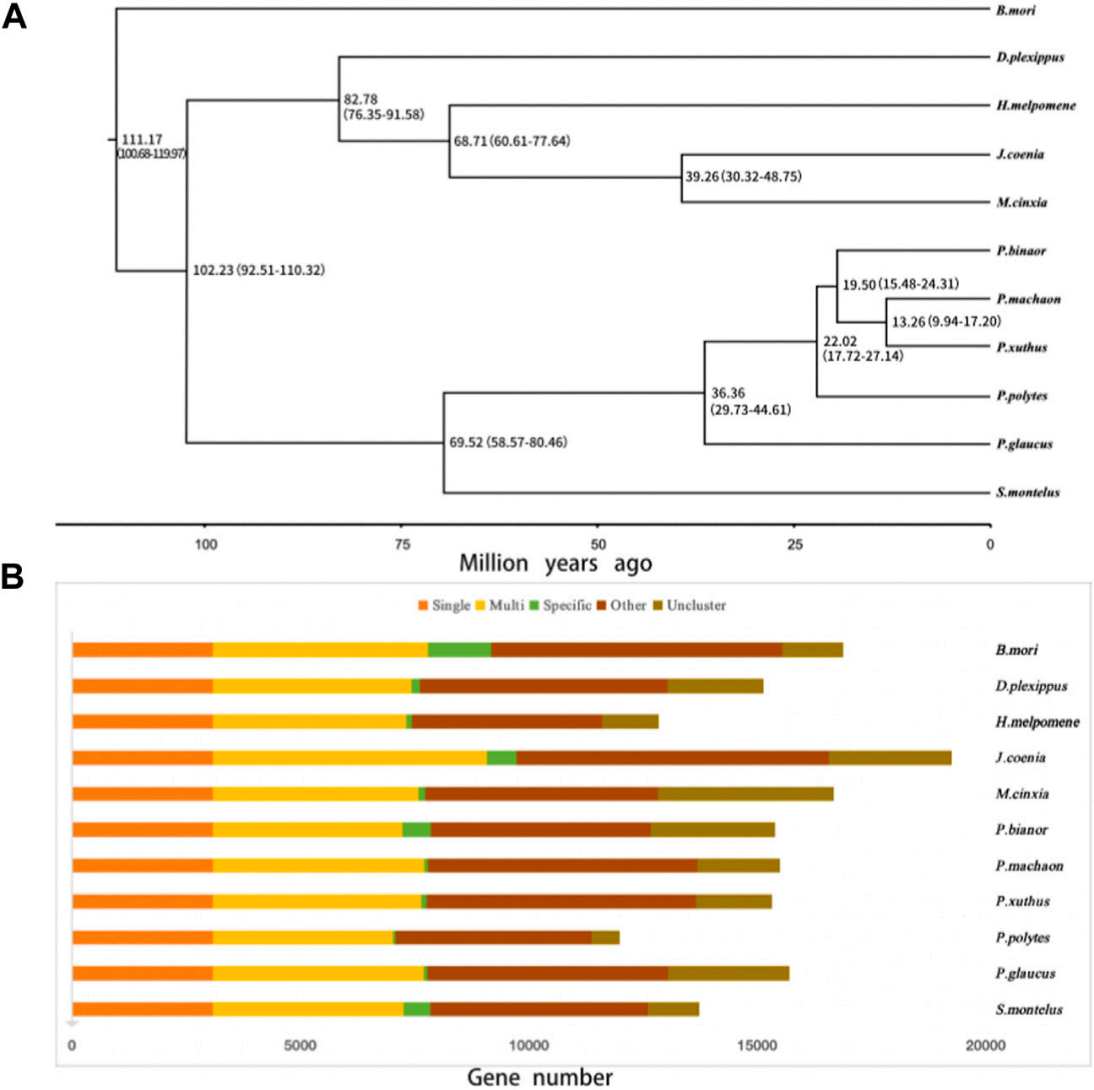
Phylogenetic tree and the orthogroup based on *S. montelus* and relative species. **(A)** Phylogenetic tree of *S. montelu*s with other species using 3071 single-copy ortholog genes. **(B)** Orthogroup between *S. montelus* and relative species. Single means single-copy orthologous genes; Multi means multiple-copy orthologous genes; Specific means genes from a unique gene family; Other means genes that do not belong to Single, Multi, or Specific; and Uncluster means unclustered genes.

**FIGURE 4:**
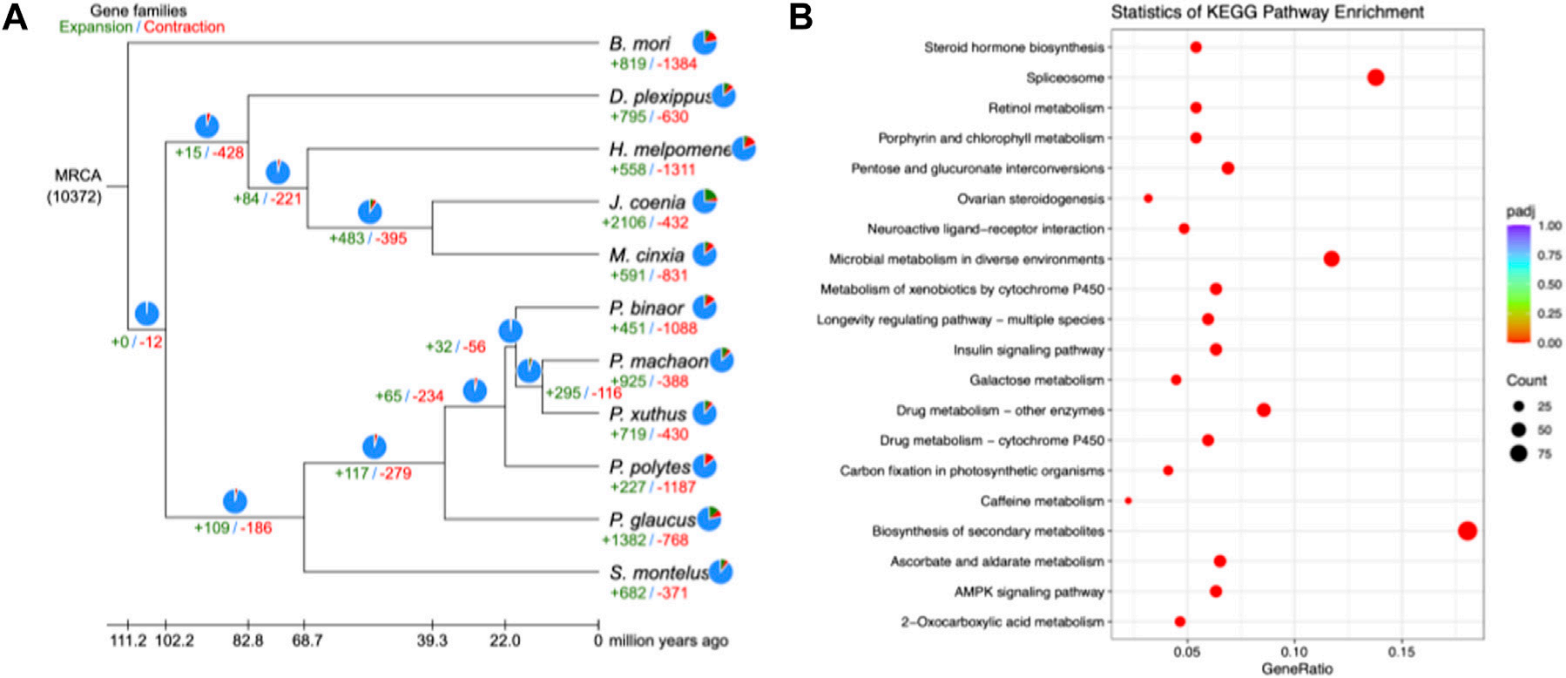
Gene family dynamics in *S. montelus* and relative species. **(A)** Expansions and contractions of gene families residing in *S. montelus* and its relatives. The green color represents expansion gene families and numbers. The red color represents contraction gene families and numbers. **(B)** Top 20 pathways with members of gene families enriched in *S. montelus.*

In the present study, 14,986 gene families were identified from the predicted protein-coding genes of *S. montelus*, and the sequences were downloaded from public databases for relative species. Of these gene families, 3071 single-copy (one-to-one) ortholog gene families were used to construct their phylogeny relationships and divergence times. The results showed that all *Papilio* species formed a monophyletic group, and *S. montelus* diverged from the common ancestor of all *Papilio* species about 58.57–80.46 million years ago (Mya) (Figure 3). All other butterflies, including *D. plexippus*, *H. melpomene*, *J. coenia*, and *M. cinxia*, formed another single clade, with the moth *B. mori* forming the basal branch, and diverged about 100.68–119.97 Mya (Figure 3). All these phylogenetic relationships were fully supported by 2000 times of ultrafast bootstrap resampling. Given the number of members for each gene family and the divergence times among these species, the gene family dynamics during evolution history were deduced. For *S. montelus*, 682 expansion gene families and 371 contraction gene families were found. The enrichment analysis about the genes related to expansion families showed that several KEGG pathways, including ascorbate and aldarate metabolism, retinol metabolism, metabolism of xenobiotics by cytochrome P450, and others, were significantly enriched (Figure 4 and Supplementary Table S7). A total of 98 cytochrome P450 genes in the *S. montelus* genome (Supplementary Table S8) and 86 cytochrome P450 genes in the *P. polytes* genome were annotated compared with those in *B*. *mori*, which is a major insect model for research (Kawamoto et al., 2019), and another common species of swallowtail butterfly *P. polytes,* which does not feed on *Aristolochia* plants. Cytochrome P450 is a large superfamily of heme-thiolate proteins, including clan2, clan3, clan4, and clan mitochondrial subtypes (https://kaikobase.dna.affrc.go.jp/curation/P450.html), in the *S. montelus* genome (Fig. S5). In the present study, clan3 showed a large expansion (Table 5). P450 expansions mainly occurred from gene duplication (Cheng et al., 2017). The large P450 cluster from clan3, which contained nine CYP332A1 genes, was located in a cluster on the same chromosome (Supplementary Figure S6).

**TABLE 5:** Comparison of cytochrome P450 subgene families between *S. montelus* and *Bombyx mori*.

Family	Clan	*S. montelus*	*P. polytes*	*B. mori*
P450	Clan 3	45	31	32
	Clan 4	34	36	33
	Mitochondrial	11	10	11
	Clan 2	8	9	7
	Total	98	86	83

The P450 enzymes are a diverse class of genes that play important roles in the metabolism of endogenous and foreign chemicals (Feyereisen, 1999). Thus, the members of the insect P450 superfamily may participate in the adaptation processes, including insecticide resistance and tolerance to plant toxins (Scott et al., 1998). In this sense, AAs have been demonstrated to be highly correlated with aristolochic acid nephropathy and even associated urothelial malignancies; in mammals such as mice and rats, hepatic cytochrome P450s may contribute to the detoxication of these plant toxins (Stiborova et al., 2011; Luan et al., 2015). Therefore, given that the larvae of *S. montelus* mainly fed on *Aristolochia*, the significant expansion of P450 members in this species to increase the tolerance level was not unexpected. The immunogold signals showed that AAs were observed all over the cells of mouse renal tubules and accumulated in the mitochondria and peroxisomes (Li et al., 2014). Thus, the significant expansion of genes related to peroxisomes might help attenuate the toxic effects of AAs. The impact of AAs on other pathways, such as galactose metabolism (Nie et al., 2015; Hu et al., 2017), caffeine metabolism (Nie et al., 2015), and MAPK signaling pathway (Chen et al., 2019), have also been reported. Although the molecular mechanisms were not well known, it was hypothesized that the expansion of family members associated with these pathways might be related to AA tolerance. For transcriptome analysis, mRNAs in different stages (egg, larva, pupa, and adult), including P450 gene families, were quantified. The results showed that 47 of 98 P450 genes had a higher expression level in the larval stage compared with the other stages (Supplementary Figure S7). Of the 47 higher-expression genes, 37 were significantly overexpressed, including 19 CYP3 clan genes, 10 CYP4 clan genes, 5 mitochondrial clan P450 genes, and 3 CYP2 clan genes.

Subsequently, a positive selection analysis was conducted. Positive selection is the process by which new advantageous genetic variants sweep a population. Thus, possible amino-acid adaptations residing in the protein-coding genes within the *S. montelus* genome were identified. Using the best BLAST hit strategy, 13,081 possible ortholog groups were found among these species. By adopting the branch-site model with 8474 orthologous genes presented in *S. montelus*, 115 of these orthologous genes were identified to be probably subject to positive selection during the adaptation of *S. montelus* (Supplementary Table S9). The functional analysis showed that these genes, including *ppib* (peptidyl-prolyl *cis*-*trans* isomerase B), *qtrt1* (queuine tRNA-ribosyltransferase), and *rps4* (small subunit ribosomal protein S4e), might participate in several KEGG pathways, such as Notch signaling pathway, Toll and Imd signaling pathway, and amino sugar and nucleotide sugar metabolism. However, the enrichment analysis showed that no other pathways were significantly enriched (Supplementary Table S10).

Several genes in the list of 115 elements under positive selection were reported to be involved in AA exposure in previous studies on mammals. The elongin A (*eloa*) and HEAT repeat containing 1 (*heatr1*) could be altered by the overexposure of human renal epithelial cells to AAs (Li et al., 2021). In this case, the expression levels of *eloa* increased after treatment with AA, unlike *heatr1*, which resulted in an underexpression. Moreover, in the same study, the expression level of *aph1b*, which should be one of the homologous genes of gamma-secretase subunit APH-1A (*aph1a*), also increased. Therefore, these genes may be related to the interactions between the organism and AAs (Li et al., 2021). The directional alterations in the metabolism of some amino acids may impact the efficacy of promoting tolerance to AAs (Stepka et al., 2021). For example, the eukaryotic transcription process mediated by RNA polymerase II (pol II) may be paused by several situations such as DNA damage (Van Houten and Kisker, 2014). In this case, the elongin complex can mediate the ubiquitylation and degradation of the largest subunit of pol II (Rpb1) to stimulate transcription elongation (Yasukawa et al., 2008). The elongin is composed of two small regulatory B and C subunits and a transcriptionally active A subunit, which is coded by *eloa*. *In vivo*, AAs can be reduced to an electrophilic cyclic N-acylnitrenium ion and preferentially form DNA adducts and cause damage (Schmeiser et al., 2009). Therefore, it was presumed that the modifications of two amino acids in the coil regions of the A subunit (Supplementary Figure S8) might promote the efficacy of the elongin and accelerate the DNA-damage-induced ubiquitylation and subsequent degradation of the Rpb1 complex. The γ-secretase complex participated in the cleavage of amyloid precursor protein into amyloid beta-peptide (Aβ), and the protein Aph1 encoded by *aph1* was an important scaffold protein in the γ-secretase complex (Watanabe et al., 2022). The functional analysis showed that the γ-secretase complex containing Aph1b produced more Aβ42 than the complex containing Aph1a (Serneels et al., 2009), and the Aph1 mutant analysis suggested the regulatory function of Aph1 in γ-secretase activity (Watanabe et al., 2022). Two mutations within the helix regions of Aph1a detected in *S. montelus* might enhance the catalytic activity of the γ-secretase complex (Supplementary Figure S8), although the adaptative mechanisms in AA endurance are yet unknown. Heart1 may act as an integrative hub of pre-rRNA transcription and processing (Prieto and McStay, 2007). The downregulation of *heatr1* can lead to the increased expression of p53 (Turi et al., 2018), which is a critical gene in carcinogenesis and frequently mutated in AA-induced urothelial tumors (Slade et al., 2009). It was hypothesized that positive selection might alter the efficiency of the genes and lead to relatively stable p53 expression levels in *S. montelus*, as the activation of p53 might promote renal injury in acute AA nephropathy (Zhou et al., 2010). However, these regulatory relationships were only testified in mammals. The actual molecular events in insects, such as *S. montelus*, warrant further investigations.

## Conclusion

This study reported a high-quality ornamental butterfly genome *S. montelus* with Nanopore long reads sequencing and Hi-C scaffolding technologies. The relationships and divergence times between *S. montelus* and relative species were identified based on the *de novo* assembly. *S. montelus* was separated from the common ancestor of swallowtails about 58.57–80.46 Mya. A comparison of the protein-coding genes in the species and relatives showed that a series of adaptations might happen in the *S. montelus* genome. The gene family expansion and expression might highlight this as an area for future research on the adaptation to the feeding habits of the larval phase of the species. Three of the 115 genes in previous studies with AA tolerance in mammals were positively selected in *S. montelus.* Thus, this study provided not only novel biological insights but also useful butterfly data for further genetic and comparative genomics analyses with this species or closely related ones.

## Data Availability

The datasets presented in this study can be found in online repositories. The names of the repository/repositories and accession number(s) can be found in the article/Supplementary Material.
