# Supplementary material for: De novo assembly of a chromosome-level reference genome of the ornamental butterfly Sericinus montelus based on nanopore sequencing and Hi-C analysis
Source: Front Genet. 2023 Mar 8;14:1107353. doi: 10.3389/fgene.2023.1107353 (PMC10030965; doi:10.3389/fgene.2023.1107353)
Supplement: Supplementary file 1 [file DataSheet1.docx]

Supplemental Information for:

*De Novo* Assembly of a Chromosome-Level Reference Genome of the Ornamental Butterfly *Sericinus Montelus* Based on Nanopore Sequencing and Hi-C analysis

# Supplemental Figure


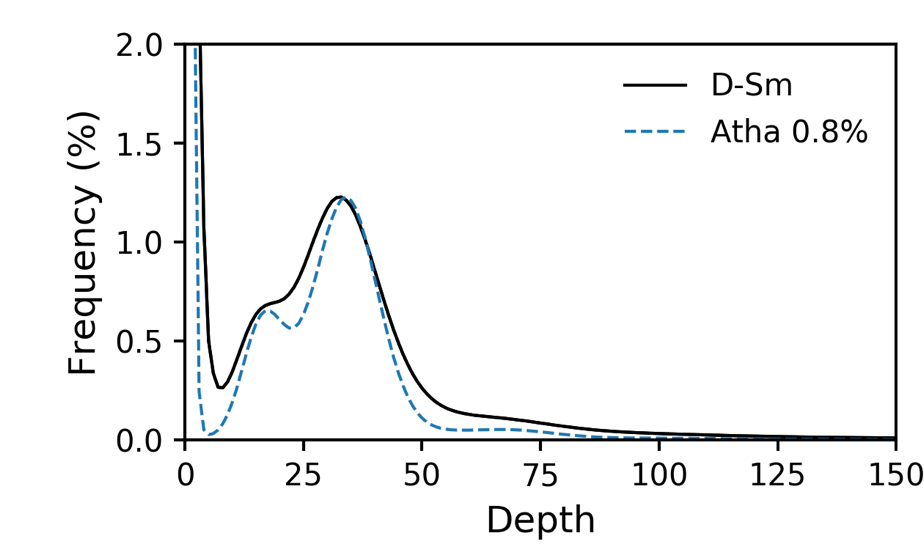


**Supplemental Figure** **S1**. Distribution of 17-mers calculated using short reads of whole genome sequencing for *S. montelus*. The 17-Kmer peak depth was 33 in the distribution.D-Sm is the sample name of *S.montelus*,Atha is simulate data form *A.thaliana*


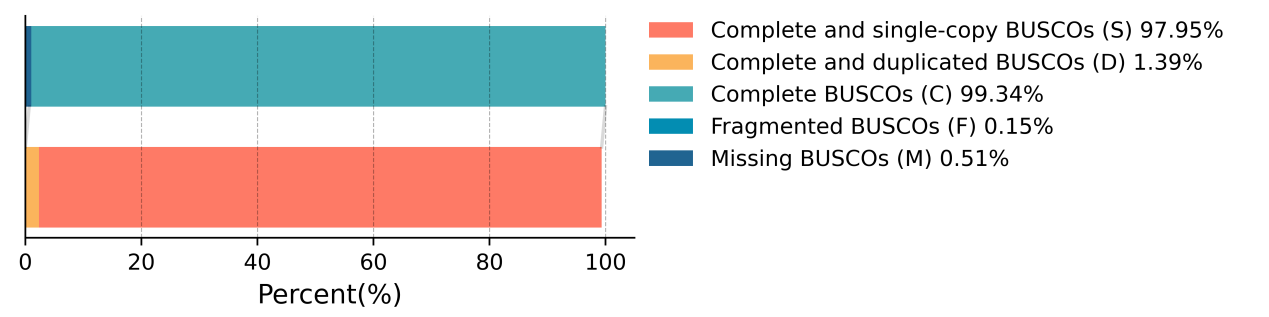


**Supplemental Figure S2.** Genome assembly BUSCO evaluation.


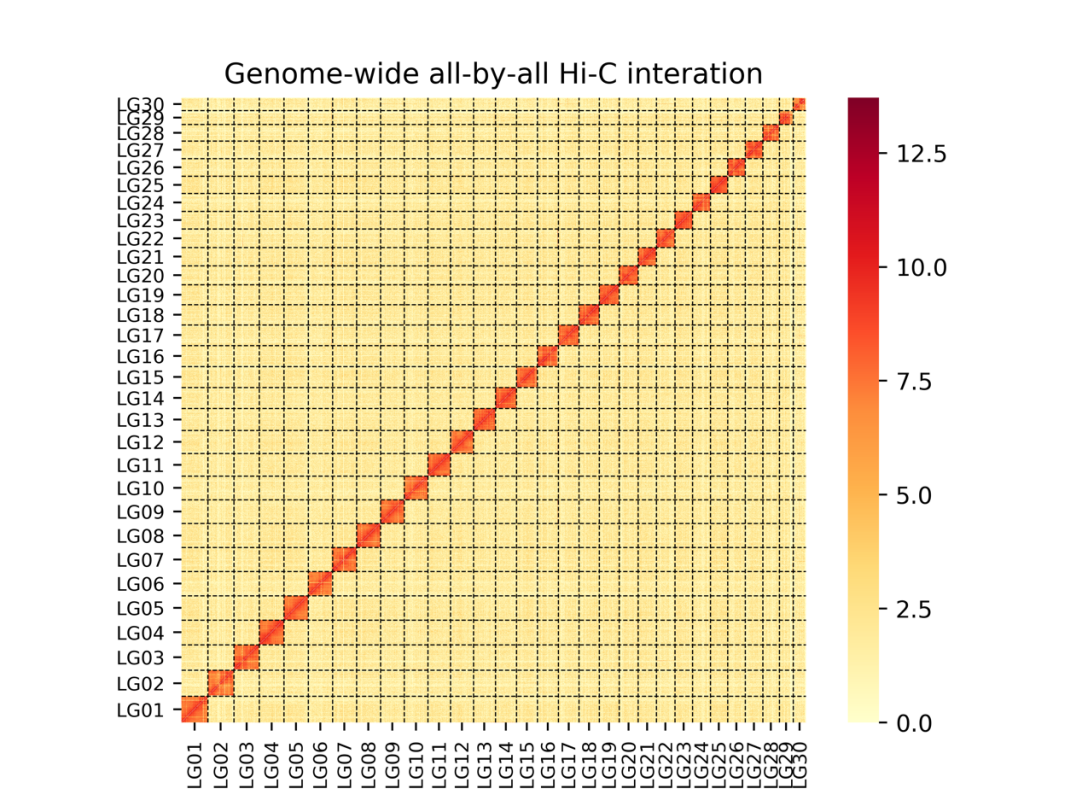


**Supplemental Figure S3**. Interchromosomal Hi-C contact matrix with 100-kb windows on 30 chromosomes.


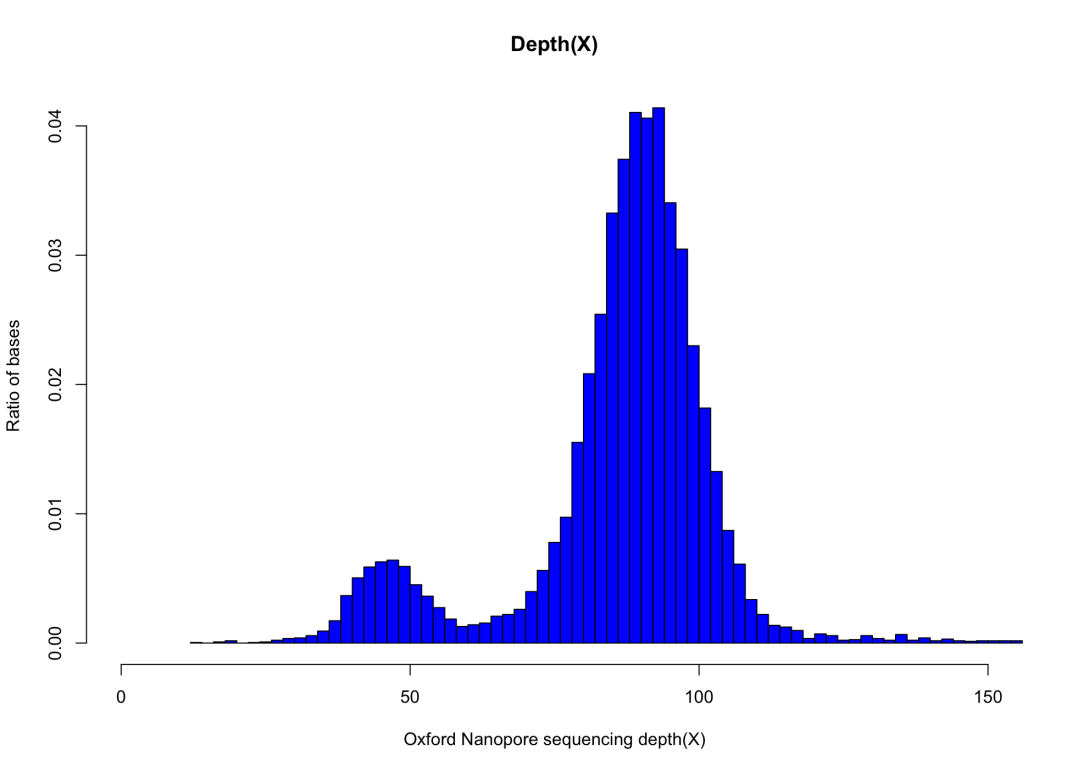


**Supplemental Figure S4**. Sequencing depth across the assembled genome with Oxford Nanopore data.


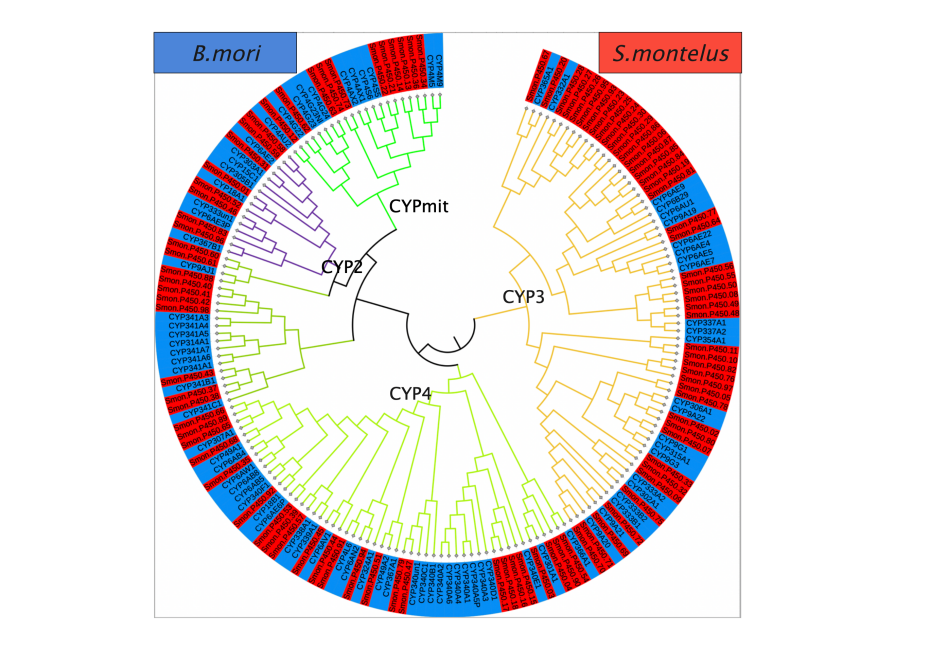


**Supplemental Figure S5**. Comparison of P450 gene family between *B.mori* and *S.montelus*


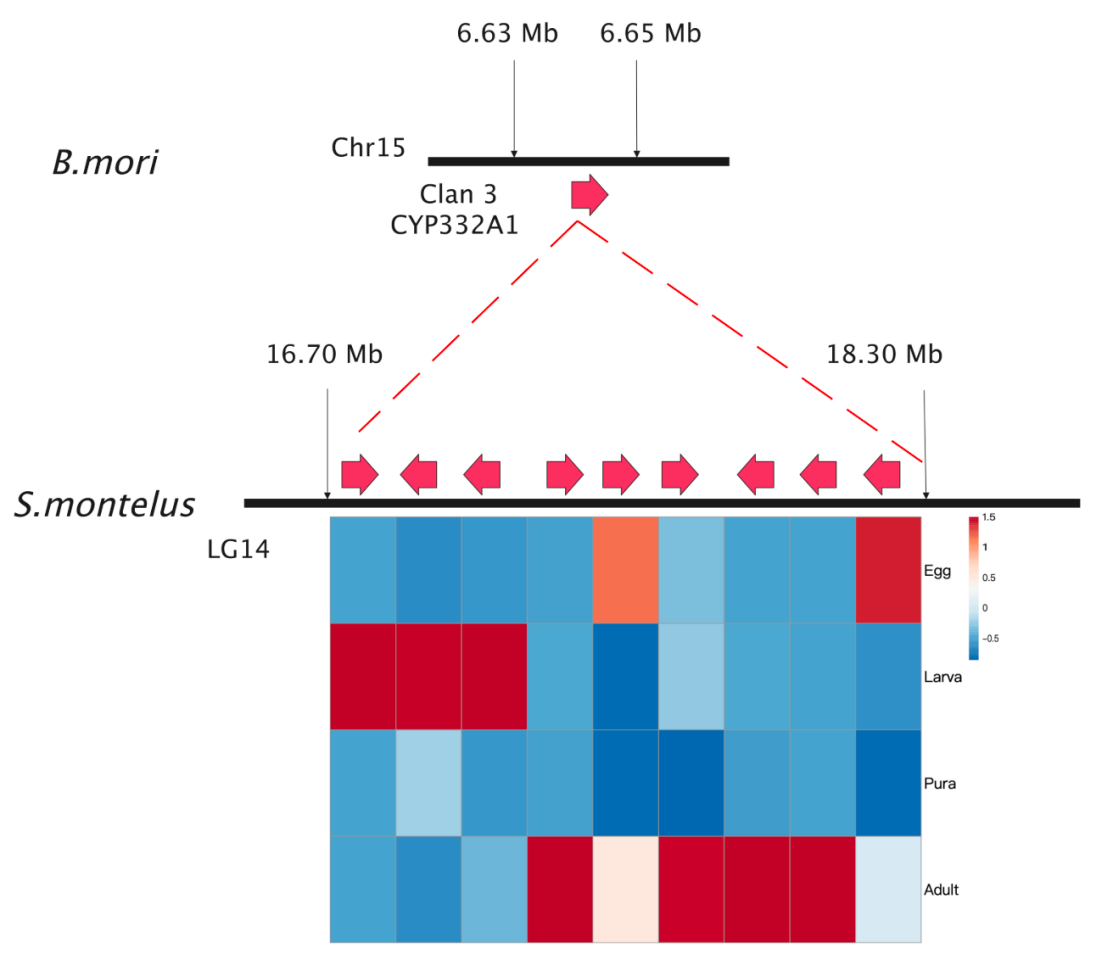


**Supplemental Figure S6**. Large expansion and expression of P450 Clan3 gene CYP332A1 on LG14.


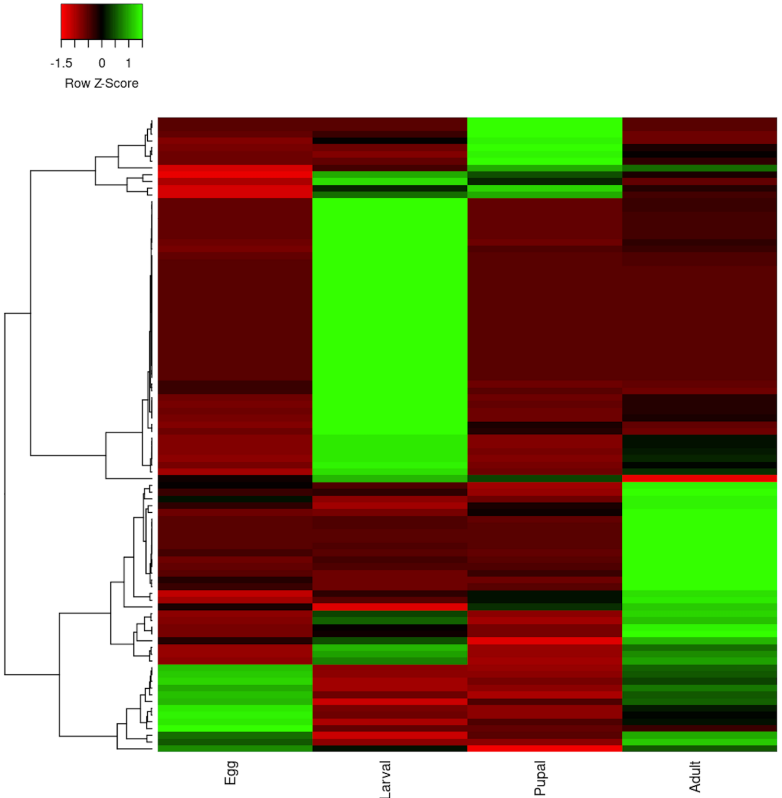


**Supplemental Figure S7**. Gene expression of the P450 gene family in different stages .


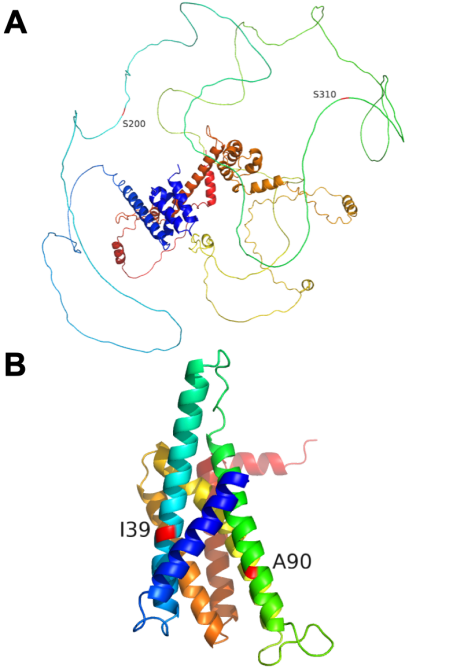


**Supplemental Figure S8**. The predicted 3D structures of two candidate genes probably subject to positive selection. Possible sites of adaptative modifications were marked in red and labeled. A: 3D structures elongin A. B: 3D structures of gamma-secretase subunit APH-1A.
